# The role of vitamin D-synthesizing enzyme CYP27B1 in systemic lupus erythematosus

**DOI:** 10.55730/1300-0144.5399

**Published:** 2022-03-26

**Authors:** Man LUO, Jing LIU, Yeshuang YUAN, Yong CHEN, Guohua YUAN

**Affiliations:** 1 Division of Rheumatology, Department of Internal Medicine, Suining Central Hospital, Sichuan, China; 2 Division of Institute Rheumatology, Department of Internal Medicine, Faculty of Medicine, Affiliated Hospital of North Sichuan Medical College, Sichuan, China; 3 Division of Rheumatology, Department of Internal Medicine, Jianyang People’s Hospital, Sichuan, China

**Keywords:** Systemic lupus erythematosus, 1α-hydroxylase, vitamin D

## Abstract

**Background/aim:**

To measure the expression of 1α-hydroxylase (CYP27B1) and serum 25(OH)D concentration in systemic lupus erythematosus (SLE) and to investigate the role of CYP27B1 in SLE.

**Materials and methods:**

Seventy-seven SLE patients and 35 healthy controls (HCs) were enrolled from September 2017 to January 2020. The study design is cross-sectional. mRNA expression of CYP27B1 in peripheral blood mononuclear cells (PBMCs) was measured by reverse-transcription quantitative PCR, the protein level of CYP27B1 was quantified by western blotting, and the serum level of 25(OH)D was determined by an enzyme-linked immunosorbent assay.

**Results:**

The mRNA expression of CYP27B1 in PBMCs was significantly lower in SLE patients than in HCs (*p* < 0.001), and the protein quantification confirmed that CYP27B1 expression was lower in SLE patients than in HCs (*p =* 0.001). Among SLE patients, the prevalence of lupus nephritis was higher in a subgroup with lower CYP27B1 mRNA expression than in a subgroup with normal CYP27B1 mRNA expression (41.07% *vs*. 14.28%, *p =* 0.028). The mRNA expression of CYP27B1 negatively correlated with the Systemic Lupus Erythematosus Disease Activity Index (*r* = −0.331, *p =* 0.003). Serum 25(OH)D concentration was lower in SLE patients than in HCs (37.64 ± 19.89 *vs*. 50.58 ± 12.74 ng/mL, mean ± SD, *p =* 0.003).

**Conclusion:**

The expression of CYP27B1 in PBMCs may be related to SLE pathogenesis, disease activity, and nephritis.

## 1. Introduction

Systemic lupus erythematosus (SLE) is an autoimmune disease involving multiple organs. It is characterized by the emergence of autoantibodies, a reduction in the number of regulatory T cells (Tregs) or a loss of their function, and excessive proliferation of B cells [1]. An environment, genes, viruses, and other factors can affect the initiation and development of SLE. Vitamin D is one of the environmental factors. Extrarenal transformation of 25(OH)D to 1,25(OH)_2_D requires mitochondrial enzyme 1α-hydroxylase (CYP27B1), and a variety of immune cells including monocytes/ macrophages, dendritic cells, B lymphocytes, and T lymphocytes express CYP27B1 and can convert 25(OH)D into 1,25(OH)_2_D. CYP27B1 expression and activity in immune cells (in contrast to kidneys) can be regulated by immune signals. Circulating 25(OH)D forms a complex with vitamin D-binding protein (DBP) and reaches target cells, such as immune cells. CYP27B1 in the target cells hydroxylates 25(OH)D to 1,25(OH)_2_D [2]. The latter maintains the balance between Tregs and effector T cells by increasing the number of Tregs and reducing that of T helper 1 (Th1) cells and Th17 cells, diminishes memory B-cell counts and antibody levels, and inhibits the expression of some inflammatory factors [1]. This paper addresses the role of the vitamin D-synthesizing enzyme CYP27B1 in SLE.

## 2. Materials and methods

### 2.1. Samples

Seventy-seven outpatients and inpatients with SLE in the Department of Rheumatology and Immunology of the Affiliated Hospital of North Sichuan Medical College were enrolled from September 2017 to January 2020, including 4 males and 73 females, aged 16 to 70 years (37.76 ± 12.14 years). The exclusion criteria for the SLE patients were infection, a tumor, liver or renal dysfunction, diabetes, hypertension, pregnancy, malnutrition, and non-SLE immune diseases. Inclusion criteria were as follows: subjects not taking vitamin D or analogues for more than 1 month, and (for SLE patients) subjects meeting the diagnostic classification criteria formulated by the American College of Rheumatology (ACR) in 1997 [3]. The disease activity was evaluated by means of the Systemic Lupus Erythematosus Disease Activity Index (SLEDAI). Sex- and age-matched 35 healthy controls (HCs) during the same season were selected. The study protocol complied with ethics guidelines and was approved by the ethics committee of the Affiliated Hospital of North Sichuan Medical College. The subjects provided informed consent and signed an informed consent form.

### 2.2. Methods

#### 2.2.1. Isolation of peripheral blood mononuclear cells (PBMCs)

Peripheral venous blood (5 mL) was collected from each subject into a tube containing ethylenediaminetetraacetic acid (EDTA). PBMCs were isolated by density gradient centrifugation (2000 *g*) with a lymphocyte-separating solution (Tianjin Hao Yang, Biological Products Technology Co.,Ltd.), according to the manufacturer’s instructions.

#### 2.2.2. Reverse-transcription quantitative PCR (RT-qPCR)

Total RNA was extracted by means of TRIzol (Ambion, Austin, TX, USA) according to the manufacturer’s instructions and was dissolved in RNase-free deionized water. The purity and concentration of RNA were determined on an ultraviolet spectrophotometer, and the RNA was reverse-transcribed into cDNA using the QuantiTect Reverse Transcription kit (Qiagen, Hilden, Germany), according to the manufacturer’s instructions. The following primers were used: CYP27B1, forward 5′-CCA TGT GGC AGA AGG GAT AA-3′, reverse 5′-AAA CCG TAA ACC AGG CTA GG-3′; and β-actin, forward 5′-CAT CAC GAT GCC AGT GGT ACG-3′, reverse 5′-AAC CGC GAG AAG ATG ACC CAG-3′. They were synthesized by the Bioneer company (Korea). The SYBR Green Real-time Quantitative PCR Kit (Qiagen, Hilden, Germany) was used, and the total reaction volume (20 μL) comprised double-distilled H_2_O (7.1 μL), SYBR Green (10.1 μL), a forward primer (0.4 μL, 10 μM), a reverse primer (0.4 μL, 10 μM), and cDNA (2 μL). The PCR conditions were as follows: 95 °C for 2 min, 95 °C for 5 s, and 60 °C for 14 s, for a total of 40 cycles. The reaction was carried out on a Quant Studio 12K Flex Real-time PCR instrument (ABI, USA). All the samples were assayed as three technical replicates. There was no template control. The expression of CYP27B1 is presented as an RQ value. The larger the RQ value, the higher is relative expression of CYP27B1.

#### 2.2.3. Western blotting

Total protein was extracted from PBMCs of SLE patients and HCs, and protein concentration was determined by the bicinchoninic acid assay. In western blots, the primary antibody was a rabbit monoclonal anti-CYP27B1 antibody (Abcam, UK), and the secondary antibody was a horseradish peroxidase-conjugated antirabbit IgG antibody (Proteintech, USA); a rabbit polyclonal anti-GAPDH antibody (BODE Bioengineering Co., Ltd., China) was used to set up an internal reference. Protein expression of CYP27B1 in PBMCs was assessed by western blotting. The amount of total protein loaded on the gel was 40 **μ**g per lane, and the PAGE Gel Rapid Preparation Kit (10%) was employed (Shanghai Ya Mei Biotechnology Co., Ltd., China) for the electrophoresis. After that, the proteins were transferred to a membrane. The latter was blocked at 4 °C on a shaking table and then incubated overnight (anti-CYP27B1 antibody dilution 1:1000, anti-GAPDH antibody dilution 1:5000) at room temperature with shaking for 1 h. Next, the membrane was probed with the secondary antibody (dilution 1:5000). After washing, the membrane was incubated with an electrochemiluminescence (ECL) reagent for color development, followed by exposure and imaging. The ImageJ software was used for densitometry of the protein bands.

### 2.3. Determination of serum 25(OH)D concentration by an enzyme-linked immunosorbent assay

This procedure was performed in the Laboratory Department of the Affiliated Hospital of North Sichuan Medical College with reagents from Roche (Germany).

### 2.4. Statistical methods

Statistical analysis was performed in SPSS version 22.0 (IBM, USA). Continuous variables were compared between groups of independent samples by Student’s *t* test for normally distributed data and by the Mann–Whitney test for data with a nonnormal distribution. Fisher’s exact test was carried out at *t* < 1 or 1 < *t* < 5. Spearman’s rank correlation analysis was performed to evaluate pairwise correlations between variables, and data with *p <* 0.05 were considered statistically significant. The effects of various factors on the mRNA expression of CYP27B1 were analyzed using linear regression.

## 3. Results

### 3.1 RT-qPCR results

**Figure 1:**
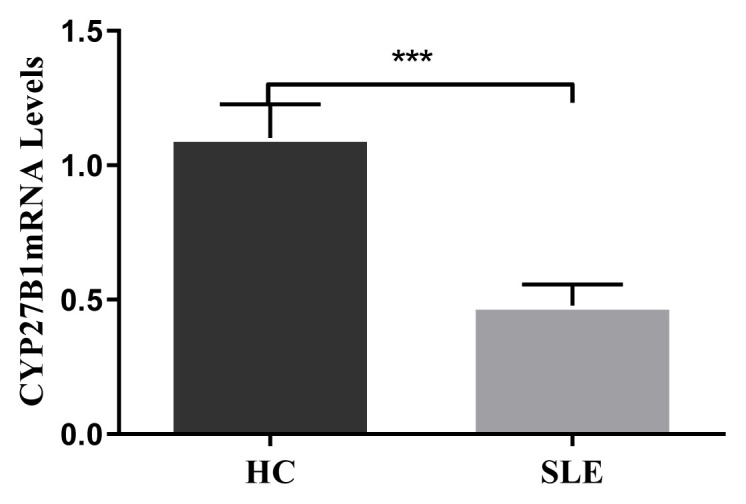
Expression of CYP27B1 mRNA in patients with HC and SLE (*p < 0.05,**p < 0.01, ***p < 0.001).

CYP27B1 is expressed in immune cells and hydroxylates 25(OH)D to 1,25(OH)_2_D, and the latter plays an immunological part [2]. The mRNA expression of CYP27B1 in PBMCs was lower in SLE patients than in HCs (*p <* 0.001) as shown in Figure 1.

### 3.2. Western blot results on CYP27B1

**Figure 2:**
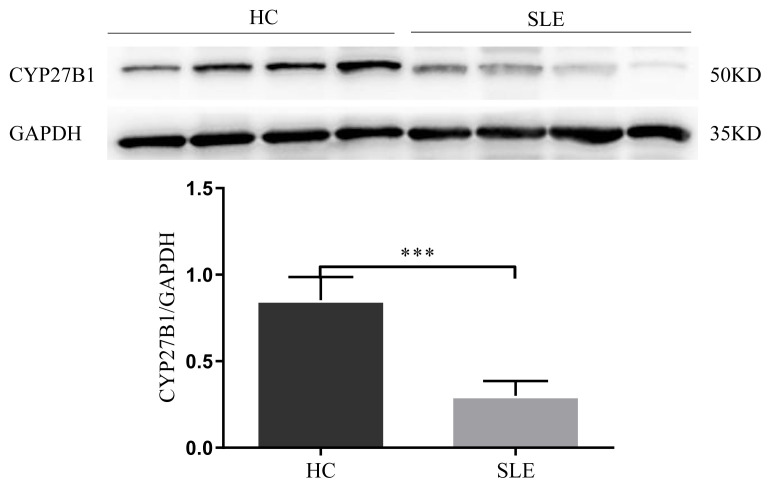
a) Western blot patterns of CYP27B1, b) expression of CYP27B1 in patients with HC and SLE (*p < 0.05,**p < 0.01,***p < 0.001).

Above, we demonstrated that the mRNA expression of CYP27B1 was lower in SLE patients than in HCs. Furthermore, the protein quantification confirmed that the expression of CYP27B1 in PBMCs was lower in SLE patients than in HCs (p = 0.001), as shown in Figures 2a and 2b.

#### 3.3.1. mRNA expression of CYP27B1 and analysis of clinical and laboratory results

We employed the median of CYP27B1 mRNA expression in the HC group as a cutoff: subjects with a lower value were assigned to a CYP27B1 mRNA low-expression subgroup, and those with a higher or equal value were assigned to a CYP27B1 mRNA normal-expression subgroup. The results of this analysis are presented in Table.

#### 3.3.2. mRNA expression of CYP27B1 after stratification by SLE phase and by steroid dose

Patients with SLEDAI < 4 were assigned to a stable-SLE cohort, and those with SLEDAI ≥ 4 to an active-SLE cohort. We also distributed all the SLE patients into a steroid-free subgroup, a low-dose steroid subgroup (prednisone acetate < 10 mg/day), a medium-dose steroid subgroup (10 mg ≤ prednisone acetate <30 mg), and a high-dose steroid subgroup (prednisone acetate ≥ 30 mg).

There was no significant difference in the mRNA expression of CYP27B1 between the no-steroid and low-dose steroid subgroups (*t* = 1.997, *p =* 0.053), between the no-steroid and medium-dose steroid subgroups (*t* = 1.94, *p =* 0.847), and between the low-dose steroid and medium-dose steroid subgroups (*F* = 0.723, *p =* 0.485) in the stable-disease cohort, and between the medium-dose steroid and high-dose steroid subgroups (*t* = 0.825, *p =* 0.417) in the active-SLE cohort. The mRNA expression of CYP27B1 was lower in the active-SLE cohort than in the stable-SLE cohort (*p <* 0.001).

### 3.4. Correlation between CYP27B1 mRNA expression and SLEDAI scores

mRNA expression of CYP27B1 among SLE patients correlated with the SLEDAI score negatively (*r* = −0.331, *p =* 0.003). The more active the disease, the lower was the mRNA expression of CYP27B1.

### 3.5. Serum levels of vitamin D

**Figure 3:**
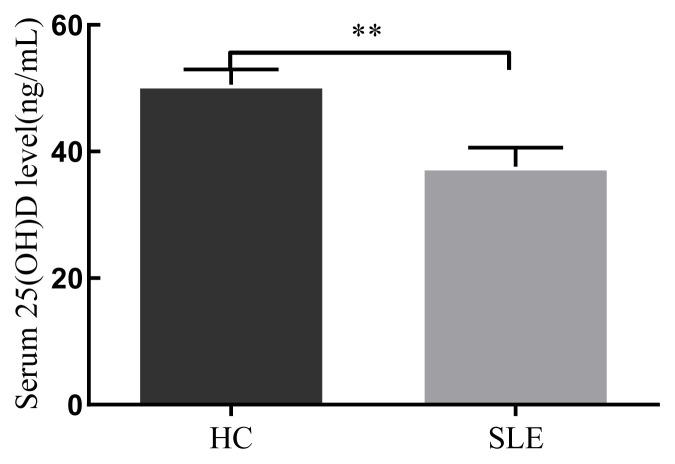
Comparison of serum 25(OH)D concentration between patients with HC and SLE (*p < 0.05,**p < 0.01).

25(OH)D is the main form of vitamin D in blood serum, and CYP27B1 can hydroxylate 25(OH)D to its active form: 1,25(OH)_2_D. The serum level of 25(OH)D was significantly lower in SLE patients than in HCs (*p =* 0.003) as shown in Figure 3.

## 4. Discussion

SLE is an autoimmune disease with multiple-organ damage. Genetic susceptibility, epidemiological risk factors, and environmental factors can affect the occurrence and development of SLE. An insufficient serum level of vitamin D may be an important factor in the pathogenesis of autoimmunity. Growing epidemiological evidence indicates that vitamin D deficiency is associated with higher incidence of autoimmune diseases. The function of vitamin D in inflammation and immune responses is mediated by nuclear vitamin D receptor (VDR) in most immune cells, including monocytes, macrophages, and activated T and B lymphocytes. 1,25(OH)_2_D_3_ can suppress NF-κB signal transduction and play an immunological role by upregulating VDR [4].

In addition to expressing VDR, many immune cells express 1α-hydroxylase (encoded by the *CYP27B1* gene), which can convert 25(OH)D into 1,25(OH)_2_D. Immune cells also express 24-hydroxylase (encoded by the *CYP24A1* gene), which is a key enzyme for inactivation of a variety of vitamin D forms. These cells can respond not only to active vitamin D metabolites but also to their precursors and have the function of vitamin D conversion [5–7]. 1,25(OH)_2_D binds to VDR to play an immunological role and to downregulate various inflammatory factors.

Some reports indicate that CYP27B1 is widely expressed in cells of many tissues and produces 1,25(OH)_2_D_3_ locally, which acts in an autocrine or paracrine manner. 1,25(OH)_2_D_3_ strongly enhances the expression of the *CYP24A1* gene via a negative feedback loop. The CYP24A1 enzyme catalyzes the hydroxylation of 25(OH)D or 1,25(OH)_2_D at position 24 to start catabolic degradation of this vitamin and to produce inactive metabolites [8, 9]. Our study shows that mRNA and protein expression of CYP27B1 in PBMCs is significantly lower in SLE patients than in HCs. The under expression of this enzyme can lead to a reduction in the amount of locally synthesized active vitamin D, thereby weakening immune function. These phenomena probably have an impact on the initiation and development of an immunological disease.

We collected and analyzed clinical data and found that the prevalence of lupus nephritis is higher while SLE is more active in patients with low mRNA expression of CYP27B1. The expression of CYP27B1 correlated with the disease activity of SLE among the patients. We analyzed the correlation between CYP27B1 mRNA expression and SLEDAI scores. The results revealed a negative correlation, suggesting that the more active the disease, the lower is CYP27B1 expression, which can also serve as one of disease-activity indicators of SLE. After excluding the effects of immunosuppression and hydroxychloroquine sulfate, we determined whether steroid therapy affects CYP27B1 mRNA expression and revealed no significant effect. Hou [10] and other researchers [11] have reported that the expression of CYP27B1 and synthetic vitamin D levels in the placenta and decidua are lower during spontaneous abortion, resulting in some placental immune disorders.

Our work showed that serum 25(OH)D concentration is significantly lower in SLE patients than in HCs. Some studies indicate that inadequate regulation of extrarenal CYP27B1 activity makes the serum concentration of 25(OH)D the main determinant of cell 1,25(OH)_2_D concentration. Intracellular concentration of 1,25(OH)_2_D is directly linked with biological effects. In some endocrine processes, target cells use 1,25(OH)_2_D_3_ as a free ligand after its dissociation from a transporter globulin called DBP. In certain autocrine/paracrine processes, 25(OH)D binds to DBP, reaches target cells, and is locally converted to 1,25(OH)_2_D_3_ [12]. The latter acts in autocrine/paracrine mode on immune cells. A quantitative decrease of 25(OH)D in serum can decrease CYP27B1 activity in immune cells, reduce the local concentration of 1,25(OH)_2_D_3_, and attenuate the corresponding immune regulatory mechanism. Dutta C [13] and other researchers [14] have demonstrated that SLE patients with vitamin D deficiency are more likely to have high disease activity, and patients with a high SLEDAI score (>10) also have a greater risk of vitamin D deficiency.

## 5. Conclusions

Our study showed that the expression of CYP27B1 in SLE patients may be associated with disease activity. 25(OH)D levels in the serum of SLE patients were found to be low; therefore, 1,25(OH)_2_D hydroxylation by CYP27B1 in immune cells may be decreased, and the corresponding immunological process is probably impaired. The regulation of vitamin D-related enzymes in immune cells involves the synthesis of 1,25(OH)_2_D by the CYP27B1 enzyme and degradation of 1,25(OH)_2_D by CYP24A1. The expression of CYP27B1 in PBMCs proved to be lower in SLE patients than in HCs. This study has a limitation: only CYP27B1 was investigated; therefore, further research is needed to test whether the imbalance of the two enzymes is related to SLE.

## Figures and Tables

**Table:** Clinical and laboratory control results of CYP27B1 mRNA expression in patients with SLE.

Project	Low expression group (n = 56)	Normal expression group (n = 21)	p-value
Female:male	53:3	20:1	0.917^d^
Age (year)	39.59 ± 12.85	35.68 ± 9.4	0.233^b^
Course of disease (month)	56.88 ± 43.66	45.14 ± 48.89	0.312^b^
Skin lesions [n(%)]	5(8.9)	2 (9.5)	0.936^d^
Alopecia [n(%)]	2(3.57)	0 (0)	1.00^d^
Blood system damage [n(%)]	9(16.1)	2 (9.5)	0.468^d^
Arthritis [n(%)]	4(7.1)	1 (4.8)	0.708^d^
Renal damage [n(%)]	23(41.07)	3 (14.28)	0.028^d^
25(OH)D(ng/mL)	38.89 ± 15.76	33.73 ± 17.97	0.365^b^
SLEDAI[M(IQR)]	2(4)	0 (1.5)	0.028^a^
HCQ[n(%)]	48(85.7)	17 (81)	0.610^c^
Immunosuppressant [n(%)]	56(100)	21 (100)	NS

Notes: SLEDAI: Systemic lupus erythematosus disease activity score.HCQ:Hydroxychloroquine.

a Mann–Whitney test;

b Student’s *t* test;

c χ^2^ test;

d Fisher’s exact test.
